# Racial Fairness of Individual- and Community-Level Proxies of Socioeconomic Status Among Birthing Parent–Child Dyads

**DOI:** 10.1007/s40615-024-02050-9

**Published:** 2024-06-25

**Authors:** Harsimran Makkad, Amisha Saini, Erika Rasnick Manning, Qing Duan, Stephen Colegate, Cole Brokamp

**Affiliations:** 1 University of Cincinnati College of Medicine, Cincinnati, USA; 2 Cincinnati Children’s Hospital Medical Center, 3333 Burnet Ave, Cincinnati, OH 45219 USA

**Keywords:** Fairness, Community- and individual-level socioeconomic status, Residential address, Geocoding

## Abstract

**Background:**

While precision medicine algorithms can be used to improve health outcomes, concerns have been raised about racial equity and unintentional harm from encoded biases. In this study, we evaluated the fairness of using common individual- and community-level proxies of pediatric socioeconomic status (SES) such as insurance status and community deprivation index often utilized in precision medicine algorithms.

**Methods:**

Using 2012–2021 vital records obtained from the Ohio Department of Health, we geocoded and matched each residential birth address to a census tract to obtain community deprivation index. We then conducted sensitivity and specificity analyses to determine the degree of match between deprivation index, insurance status, and birthing parent education level for all, Black, and White children to assess if there were differences based on race.

**Results:**

We found that community deprivation index and insurance status fail to accurately represent individual SES, either alone or in combination. We found that deprivation index had a sensitivity of 61.2% and specificity of 74.1%, while insurance status had a higher sensitivity of 91.6% but lower specificity of 60.1%. Furthermore, these inconsistencies were race-based across all proxies evaluated, with greater sensitivities for Black children but greater specificities for White children.

**Conclusion:**

This may explain some of the racial disparities present in precision medicine algorithms that utilize SES proxies. Future studies should examine how to mitigate the biases introduced by using SES proxies, potentially by incorporating additional data on housing conditions.

## Background

Precision medicine, also called personalized medicine, involves the collection, integration, and analysis of multiple sources of health data to develop individualized insights about health and disease [1]. It has the potential to improve health outcomes by enhancing clinical decisions and treatment plans [1]. Ideally, precision medicine tools can avoid the biases inherent with the current practice of using heuristics and prior experiences to make medical decisions. In reality, however, these algorithms can recapitulate longstanding health disparities and unintentionally perpetuate unequal distribution of resources [1, 2]. There are growing concerns about racial equity in precision medicine algorithms because race is often used as a predictor even though inequities can also arise due to historical and systemic disparities between racial groups, including access to and utilization of healthcare [2, 3]. Misclassification and misprediction of disease among marginalized racial groups could exacerbate racial disparities by programming further inequities in healthcare access [1, 3]. Biases can be reflected in various stages of algorithm development, from collecting data to designing and implementing algorithms in clinical practice. Machine learning algorithms that are trained on datasets lacking racial and ethnic diversity can learn to associate certain characteristics with specific racial or ethnic groups, leading to biased predictions and recommendations. This can result in inaccurate assessments and suboptimal treatment for patients from underrepresented racial and ethnic backgrounds.

Using community-level socioeconomic status as a proxy for individual-level socioeconomic is common when conducting research using only electronic health records because individual-level socioeconomic data may not be readily available or easily obtained from electronic health records alone. However, in more detailed cohort or registry studies, additional data on individual-level socioeconomic factors like education, income, and wealth can be collected and used in combination with community-level proxies to better understand the relationship between socioeconomic position and health outcomes. The choice of defining, operationalizing, and using individual- and area-level measures ultimately depends on the research question, the available data, and the level of detail needed to address the research objectives. Regardless, systemic problems with data systems and institutional racism mean that race, income, wealth, and education are highly correlated [4, 5].

Indeed, prediction algorithms often incorporate race as a proxy for otherwise unmeasured sociodemographic and environmental conditions such as socioeconomic status (SES) and air pollution that mediate most of the racial disparities in asthma [6] and a range of child health outcomes [7]. Birthing parent education or family income are ideal measures of individual SES, but many studies are retrospective and can only access information collected at the time of the study or available in existing electronic health records. As a result, researchers often use insurance type or community-level measures of SES in precision medicine algorithms. This may cause race-based differences in the accuracy of pediatric precision medicine tools. In this study, we evaluate the accuracy and fairness of using individual- and community-level proxies of SES such as insurance status and community deprivation index.

## Methods

For this study, we obtained vital records from the Ohio Department of Health for children born in Ohio from 2012 to 2021, detailing information such as birthing parent age, child race as reported by birthing parent, insurance type, birthing parent education, and birthing parent’s residential address. This study was approved with a full board review by the Ohio Department of Health Human Subjects Institutional Review Board (IRB 00002180, protocol number 2023–10).

Because our gold standard of birthing parent education level used adult educational attainment, we excluded observations if the birthing parent age was less than 18, if insurance status or education level or deprivation index were missing, or if insurance was classified as “Other.” Insurance status was dichotomized into “private” and “non-private,” where “non-private” included those individuals on Medicaid or self-pay insurance. To perform pairwise comparisons between the different measures of SES, we dichotomized birthing parent education level into “low” (less than 12 years) and “high” (12 years or greater). We used an existing categorical measure of birth parent education that was defined as “Less than 12 years (did not complete HS),” “High school graduate or GED,” and “More than high school.” Similarly, we used a racial category designed for tabulation in the Ohio Department of Health’s Public Information Warehouse that consisted of five groups: “White,” “Black,” “Native American,” “Asian,” and “Pacific Islander/Hawaiian.” For binary comparisons, we considered “Black” and “White” racial groups in order to capture the social context of membership in a historically marginalized group, specifically with respect to housing and employment opportunities that uniquely define a person’s multifaceted socioeconomic status.

Each residential birth address was geocoded to a census tract and linked to a community deprivation index value to quantify community-level SES. Six different census tract-level measures derived from the American Community Survey (fraction of the population with income in the past 12 months below the poverty level; fraction of the population ages 25 and older who have attained an education level of at least high school graduation or GED; fraction of the population with no health insurance coverage; fraction of households receiving public assistance income, food stamps, or SNAP in the past 12 months; and fraction of houses that are vacant) were reduced via principal component analysis to a single continuous variable that ranges between 0 and 1. High deprivation index was defined using a threshold equal to the 75th percentile of 2018 tract-level deprivation indices as weighted by their populations under the age of 18 (0.43) [8].

We created tables and plotted individual- and community-level SES proxies alongside birth parent education level, both overall and by race to visually detect patterns. We compared the binary classification of SES proxies (community deprivation index and insurance status) against birth parent education level to quantify their sensitivity and specificity with respect to identifying a child with a lower socioeconomic status. Sensitivity is the fraction of children correctly identified with lower SES among all children with lower SES. Specificity is the fraction of children correctly identified with high SES among all children with high SES. Sensitivity and specificity were calculated and plotted per racial group and overall to detect differences. We also compared community material deprivation between birthing parent education level when defined as a three-level category (“Less than 12 years,” “High school graduate or GED,” “More than high school”). All data analyses were conducted in R, version 4.2.3.

## Results

Between 2012 and 2021, 1,257,391 children (93.8% of all birth records) met study inclusion criteria (Table 1). Newborns were primarily Non-Hispanic (94.4%) and White (76.0%), but 17.7% of newborns were Black and 6.3% were some other race. Most birthing parents had the highest level of education (60.2%, more than high school), but 13.0% had less than 12 years of education. Insurance status was roughly equal between private (51.1%) and non-private (48.9%) payors. Almost a third of all newborns (30.8%) were living in a community with high levels of material deprivation.

**Table 1:** Demographic characteristics of Ohio births between 2012 and 2021 as reported to Ohio Department of Health

Demographics	*N*	(%)
Sex		
Female	655,648	(48.9)
Male	685,452	(51.1)
Unknown	28	(< 0.01)
Ethnicity		
Hispanic	71,772	(5.4)
Non-Hispanic	1,265,770	(94.4)
Unknown	3586	(0.3)
Race		
Asian	42,421	(3.2)
Black	237,593	(17.7)
Native American	2538	(0.2)
Pacific Islander/Hawaiian	893	(0.1)
White	1,018,940	(76.0)
Other/unknown	38,743	(2.9)
Birthing parent age		
Under 18	20,461	(1.5)
18–29	769,225	(57.4)
30–39	520,502	(38.8)
40–49	30,668	(2.3)
50 and over	213	(< 0.01)
Unknown	59	(< 0.01)
Birthing parent education level		
Less than 12 years	174,847	(13.0)
High school graduate or GED completed	352,780	(26.3)
More than high school	807,201	(60.2)
Unknown	6300	(0.5)
Insurance status		
Medicaid	530,462	(39.6)
Private insurance	685,626	(51.1)
Self-pay	67,022	(5.0)
Other	49,000	(3.7)
Unknown	9018	(0.7)
Community deprivation index, median (25th, 75th percentile)	0.36	(0.29, 0.46)
Community deprivation index		
Low, < 0.43	927,986	(69.2)
High, ≥ 0.43	412,400	(30.8)
Unknown	742	(0.1)

**Fig. 1:**
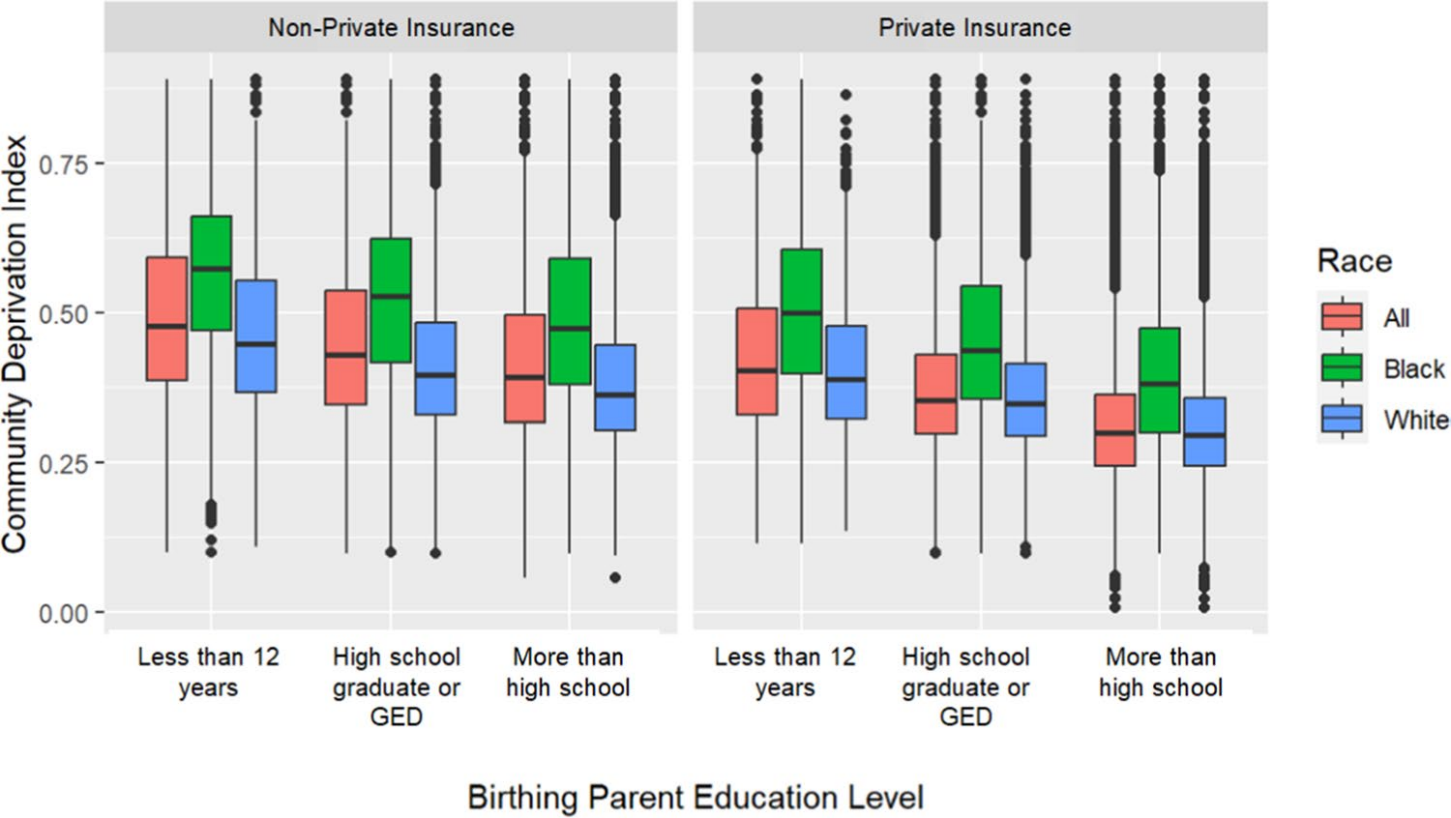
The relationship between community deprivation index, insurance status, and birth parent education level differs according to race

We first evaluated the relationships between birthing parent education level, the material community deprivation index, and non-private insurance status (Table 2). When defined as a three-level category (“Less than 12 years,” “High school graduate or GED,” “More than high school”), increasing birthing parent education level was associated with decreasing median community deprivation index among all births (0.47, 0.40, and 0.32, respectively). Although this trend is present within the subgroups of Black and White children, the median community deprivation index among Black children born to parents with more than a high school level of education (0.44) was equal to the median community deprivation index among White children born to parents with less than 12 years of education (0.44). Similarly, the fraction of births with non-private insurance decreases as birthing parent education level increases (Table 2); however, even among those with more than a high school-level education, a much larger percentage of Black children, compared to White children (62.5% versus 21.5%), had non-private insurance. Figure 1 displays the distribution of the community material deprivation index across birth parent education level when stratified by insurance status. Although community deprivation index decreases with increasing birthing parent education level and with private insurance status, Black children tend to be born into families living in communities with higher levels of material deprivation regardless of birthing parent education level or insurance status.

**Table 2:** Comparison of agreements between area- and individual-level SES proxies (i.e., community deprivation index and non-private insurance status, respectively) with SES measured via birthing parent education level overall and by race

Birthing parent education level	All children (*n* = 1,257,391)		Black children (*n* = 216,829)		White children (*n* = 963,413)	
	Median (mean) community deprivation index	% with non-private insurance	Median (mean) community deprivation index	% with non-private insurance	Median (mean) community deprivation index	% with non-private insurance
Less than 12 years	0.47 (0.49)	91.6	0.57 (0.56)	94.0	0.44 (0.46)	90.4
High school graduate or GED	0.40 (0.43)	69.7	0.51 (0.52)	86.0	0.38 (0.40)	63.5
More than high school	0.32 (0.34)	27.1	0.44 (0.45)	62.5	0.31 (0.32)	21.5

**Fig. 2:**
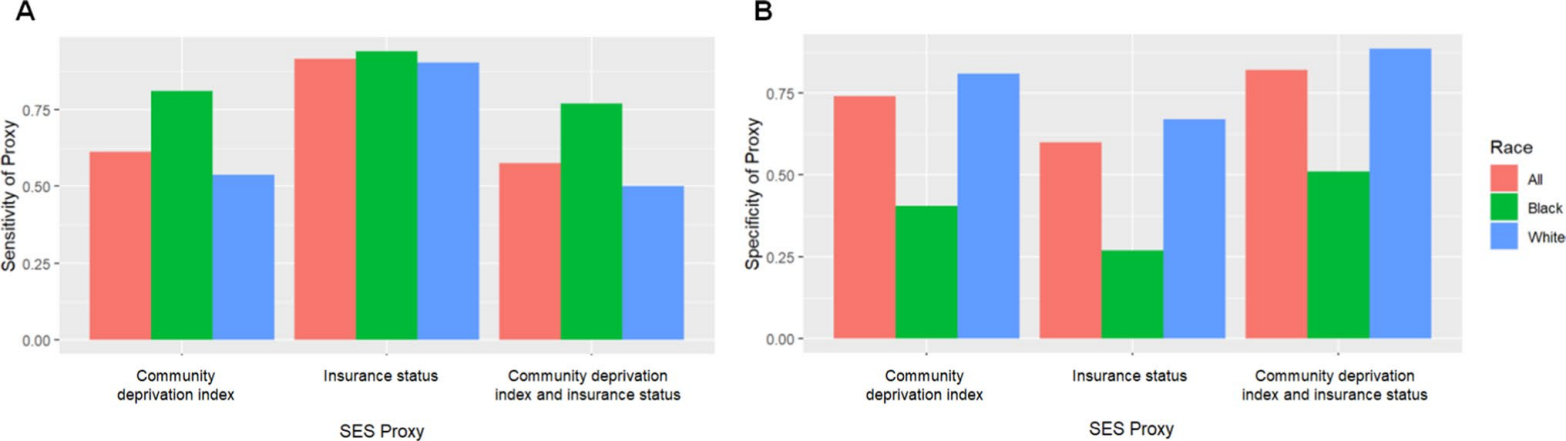
Sensitivity (**A**) and specificity (**B**) analyses of the degree of agreement between SES proxies (community deprivation index and insurance status) and birthing parent education level overall and by race

When evaluating SES proxies by racial group, they were consistently more sensitive among Black children and more specific among White children (Fig. 2). For example, the community deprivation index was more sensitive among Black children (81.1%) compared to White children (53.7%) but was less specific (40.4% versus 81.0%). Likewise, insurance status was more sensitive among Black children (94.0%) compared to White children (90.4%) but was less specific (27.0% vs 67.0%). Overall, this means that using these proxies can better identify low-SES Black children compared to White children but come with a cost of not being able to identify high-SES Black children compared to White children.

## Discussion

In this study, we found that community deprivation index and insurance status, alone or in combination, fail to accurately represent individual-level SES, as measured by birthing parent education level, and that inconsistencies differ by race. Specifically, these SES proxies tend to overestimate the amount of socioeconomic deprivation experienced by Black children as compared to White children. This means epidemiologic studies that utilize these SES proxies could be introducing exposure assessment error that is differential by race.

Because asthma is a leading cause of pediatric health disparities [9], one area of concern is racial equity in pediatric asthma prediction algorithms [10]. The goal of precision medicine in pediatric asthma is to identify children at risk earlier so that they can receive necessary preventative care. As this is contingent on accurate prediction, studies have begun investigating the reliable prediction of asthma risk to understand biological mechanisms, inform early interventions, and improve long-term respiratory outcomes [4, 11, 12]. Among the available prediction tools for childhood asthma, we previously found that racial disparities existed in three of four criteria for both the Asthma Predictive Index (API) and Pediatric Asthma Risk Score (PARS) [10]. Race is often incorporated as an approximation of not only environmental factors such as air pollution but also unmeasured sociodemographic factors such as poverty and income, which are considered as proxies for SES and therefore risk factors for the development of pediatric asthma [6, 13]. Clinically, the disparities in inaccuracies we found in this study mean that using proxies for SES will overpredict morbidity among Black children, as Black children from high SES backgrounds will more often be misclassified as low SES and therefore at higher risk. Furthermore, the misclassification of Black children from high SES backgrounds and the increased rate of false positives mean that future health outcomes for Black children who are erroneously believed to be from low SES backgrounds may appear to be less related to SES. In health policy, underestimating the relationship between SES and health outcomes among Black children may lead to redistribution of resources away from Black children truly in need.

One limitation of our study is the restriction to records from the State of Ohio, which means that our results may not be transportable to other settings or states; however, our population-level coverage of all births in Ohio means that selection biases related to our target population of children in Ohio were likely minimal. Future research could examine how to identify and mitigate bias caused by using SES proxies in epidemiologic studies. Other sources of extant data, including information about property value and tenure at an address-specific level, could be used to supplement SES information for clinical and research use [13, 14]. Additionally, there are numerous other individual SES indicators that should be tested in the future. Regardless, clinicians and researchers should recognize the challenges that arise with measuring SES using community- and individual-level proxies and interpret findings accordingly. In conclusion, considering fairness in precision medicine should be a routine part of tool development and validation in order for healthcare professionals to be able to accurately screen at-risk children and provide more personalized care, thereby helping reduce disparities in health outcomes.

## Data Availability

Vital records are housed at the Ohio Department of Health and are not available to non-study personnel.

## Code Availability

The material deprivation index is openly available at https://geomarker.io/dep_index. Code for comparing racial fairness of socioeconomic measures is available upon request.
